# Multifocus Image Fusion Using Wavelet-Domain-Based Deep CNN

**DOI:** 10.1155/2019/4179397

**Published:** 2019-02-20

**Authors:** Jinjiang Li, Genji Yuan, Hui Fan

**Affiliations:** ^1^School of Computer Science and Technology, Shandong Technology and Business University, Yantai 264005, China; ^2^Co-innovation Center of Shandong Colleges and Universities: Future Intelligent Computing, Yantai 264005, China

## Abstract

Multifocus image fusion is the merging of images of the same scene and having multiple different foci into one all-focus image. Most existing fusion algorithms extract high-frequency information by designing local filters and then adopt different fusion rules to obtain the fused images. In this paper, a wavelet is used for multiscale decomposition of the source and fusion images to obtain high-frequency and low-frequency images. To obtain clearer and complete fusion images, this paper uses a deep convolutional neural network to learn the direct mapping between the high-frequency and low-frequency images of the source and fusion images. In this paper, high-frequency and low-frequency images are used to train two convolutional networks to encode the high-frequency and low-frequency images of the source and fusion images. The experimental results show that the method proposed in this paper can obtain a satisfactory fusion image, which is superior to that obtained by some advanced image fusion algorithms in terms of both visual and objective evaluations.

## 1. Introduction

Because sensor imaging technology is affected by its imaging mode, the imaging environment, and other factors, the generated image display of the target object is one-sided and superficial; reorganizing such information can describe the object more comprehensively and in more detail. Image fusion refers to the process of fusion of multiple images of the same scene into one image according to the corresponding fusion rules [1, 2]. The resulting image is more comprehensive than that obtained using the information expressed by a single source image; the resulting image exhibits clearer vision and is more consistent with human eye and machine perception [3, 4]. Therefore, the realization of multifocus image fusion is of practical significance.

The focus range of the visible light imaging system on the target area is limited by the depth of field of the optical system. In an image generated for the same scene, only the vicinity of the focus is clear and other objects are blurred to varying degrees. Multifocus image fusion technology can fuse differently focused images to generate a single image and combine some objects or information to obtain a more accurate description. Multifocus image fusion can overcome the limitations of a single sensor in terms of spatial resolution, geometry, and spectrum to improve the reliability of image processing [5], such as via feature extraction, edge detection, object recognition, and image segmentation. Multifocus image fusion technology has been widely used in remote sensing, transportation, medical imaging [6], military, and machine vision.

At present, many spatial domain-based fusion methods exist. Using these methods, better quality fused images have been obtained. Nevertheless, image artefacts are usually present in the fusion results obtained by these classical spatial methods. In response to this problem, scholars have proposed a variety of image fusion algorithms based on spatial transformation, such as image matting [7], wizard filtering fusion [8], multiscale weighted gradient [9], and quad-tree and weighted focal length measurement [10]; these algorithms can extract the details of the original image and maintain the spatial consistency of the fusion image. However, such methods cause a block effect at the boundary of the image subblock due to windowing, which has a major influence on the quality of the fused image. The fused image obtained by the image fusion method based on the transformation domain is usually accompanied by image distortion and other phenomena. Therefore, determining a new multifocus image fusion algorithm has important theoretical significance and practical value.

The key to multifocus image fusion is to extract the information of the clear part of the two images for fusion processing. This paper uses the deep learning method to learn the direct mapping between the source image and the fused image. In this paper, the deep convolutional neural network (CNN) is used to train the clear image and its corresponding blurred image to encode the mapping. The fusion rules of multifocus images can be generated through CNN model learning. On the basis of this idea, this paper uses a wavelet transform to extract the high-frequency and low-frequency information of the image and inversely transforms the fused high-frequency and low-frequency information into the fused image. The low-frequency subband of the image contains the key features of the image, and the high-frequency subband of the image contains the detailed information of the image, which is related to the sharpness of the image. A convolutional neural network is used to learn the direct mapping between the high-frequency and low-frequency subbands of the source and fusion images, respectively [11], and obtain the fusion rules of the low-frequency and high-frequency subbands. These rules determine the high-frequency and low-frequency information of the fused image. Experiments demonstrate that the fused images obtained using the convolutional neural networks are reliable.

Overall, the primary contributions of this paper cover the following three aspects:

1. An end-to-end method based on CNN is proposed for multifocus image fusion.
2. Using wavelet multiscale characteristics, high-frequency and low-frequency information is decomposed from the image. Next, the two CNNs are separately trained to encode the high-frequency and low-frequency images separately.
3. Multifocus fused images obtained through end-to-end training are of higher quality.

The remaining paper is organized as follows. In Section 2, we introduce the related work. In Section 3, the network structure employed in this paper is discussed more in detail. In Section 4, we provide details concerning the training set, training methods, evaluation indicators, and experimental results of this paper. In Section 5, we summarize the main idea and findings of this paper.

## 2. Related Work

Multifocus image fusion can be divided into three levels: pixel-level fusion, feature-level fusion, and decision-level fusion. The pixel-level fusion involves comprehensive processing using the pixel points of an image; it is the lowest level of fusion in the three levels of fusion. More information regarding the image can be obtained through this fusion such that the image is more conducive to human eye observation or computer processing. Pixel-level image fusion methods can be summarized into two categories: image fusion methods based on the spatial domain and image fusion methods based on the transformation domain.

The image fusion method based on the spatial domain [7–10] involves selecting the pixels in the clear part of an image to form a fused image. A clear area is identified based on a certain sharpness indicator, and later, the clear blocks—which are usually obtained by window or image segmentation of a specific size—are merged in the image. To obtain subblocks of an appropriate size, Bai et al. [10] specified the use of the quad-tree method to divide images into subblocks of different sizes adaptively. Some spatial domain methods based on gradient information [12–14] have also been proposed recently.

Image fusion methods based on the transformation domain usually decompose the original image into different transformation coefficients; next, they fuse these transformation coefficients by the corresponding fusion rules and finally obtain the fusion image by reconstruction of the fusion coefficient. With the development of multiscale theory, multiscale transformation (MST) has been widely applied in image fusion, including pyramid decomposition [15], discrete wavelet transform [16], double-tree complex wavelet transform [17], and nonsampled contour wave transform [18]. The basic concept of these methods is to perform multiscale decomposition on each source image, to subsequently fuse all the decomposition coefficients, and finally to reconstruct the fused image through inverse transformation. The method of combining the decomposition coefficients plays a key role in the MST-based image fusion method [19, 20]. These methods all use the same framework, which consists of decomposition [21], fusion, and reconstruction.

The spatial-domain-based approach has the advantage of directly fusing the focal region of the source image; however, this method is highly dependent on the choice of clear measurement criteria, such as gradient energy, standard deviation, or spatial frequency of the image. Since the structure information cannot be represented by a single pixel, the spatial-domain-based method requires efficient extraction of the focus area from the source image. Li et al. [7] used the matting technique to obtain the focus area of each source image. However, due to the unstable performance of the matting technique, the boundary of the focal region obtained by this method is not completely reliable. Considering the grey-scale similarity and set similarity of adjacent pixels, Kumar and Processing [22] proposed the use of a cross-bilateral filter to fuse multifocus images. However, the universality of this technique is not satisfactory, and the size of the filtering window in this method cannot be adjusted adaptively.

Some new image fusion methods, such as the method based on sparse representation (SR) [23, 24], the method based on variational and partial differential equations [25–27], and the method based on dictionary learning [6, 21], have attracted increasing attention. These methods overcome the block effect of image fusion, but the result of fusion is unstable and the edge is not natural. Zhang and Levine [23] proposed a robust sparse representation model (RSR) and multitask robust sparse representation model (MRSR). In contrast to that in the traditional SR model, the reconstruction error obtained by the decomposition of MRSR serves as the discrimination basis for the image focus region, and the focus region obtained is more accurate. However, this method uses a single source image to build a dictionary, which can easily lead to the formulation of an incomplete dictionary. Guorong et al. [28] introduced a structure tensor (ST) into image fusion to enhance the visualization of images. Li et al. [6] incorporated low rank and sparse regularization terms into the dictionary-learning model, which can effectively remove image noise and preserve texture details when merging images.

To further improve the fusion rules, many new methods have been proposed. Guorong et al. [28] and Zhao et al. [29] proposed a new transformation domain, and Li et al. [19] and Liu et al. [20] proposed a new fusion rule. Liu and Wang [30] proposed a new sparse model and more complex fusion rules. Bai et al. [10] proposed a new method of molecular block division. The existing multifocus image fusion algorithms, in particular, the image fusion algorithm based on the spatial domain, focus on proposing a new model, designing more complex fusion rules, or obtaining an index to measure the resolution of image pixels or subblocks for guiding image fusion. However, a single image feature cannot be applied suitably to a variety of complex image environments, and it is almost impossible to design an ideal fusion model that considers all factors.

Liu et al. [31] used a deep neural network for multifocus image fusion; however, the designed network is basically a classification network, which may lead to an inaccurate boundary between the focused and unfocused regions. Du and Gao [32] stated that a decision graph contains complete and clear information of the image to be fused and proposed a new multifocus image fusion algorithm based on image segmentation. A convolutional neural network was used to analyse the input image at multiple scales, and the corresponding decision graph was derived by segmenting the focus and nonfocus regions of the source image. Zhao et al. [33] proposed the use of a multilevel deep supervised convolutional neural network for multifocus image fusion and the design of an end-to-end network, through which joint generation feature extraction, fusion rules, and image fusion could be learned. Zhao et al. [33] constructed a new model to fuse the captured low-frequency features with high-frequency features. In this paper, the characteristics of a wavelet multiscale were used to decompose the image to obtain its low-frequency and high-frequency information. Xu et al. [34] attempted to use images with different foci for end-to-end mapping and establish many-to-one mapping between the source and output images. A full convolutional dual-stream network architecture was designed to realize pixel-level image fusion. Mingrui et al. [35] designed a pixel-by-pixel convolutional neural network to recognize the focus and defocus pixels in the source image for multifocus image fusion according to the neighbourhood information. However, more labels need to be designed for the focus area. In this paper, the performance of deep networks could be improved by using wavelet transform. Literature [36] proposed a residual network based on directional wavelet transform domain for low-dose X-ray CT reconstruction. The direction wavelet was used to embed the input dataset and label dataset into the high-dimensional feature space and learn its mapping. Literature [37] proposed a wavelet-based CNN multi-scale face super-resolution network, which could obtain finer details of high-resolution images. Literature [38] combined multifocus image fusion and super-resolution and used CNN to directly produce both super-resolution and full-focus output images, to obtain detailed enhanced fusion images. The considered network structure was similar to that in literature [34], but the fusion rule designed in [38] involved directly using the weight fusion, which may not achieve the ideal fusion effect. Literature [14] proposed a new multifocus image fusion algorithm based on the boundary. The focus detection task was considered to find the boundary between the focused and nonfocused regions in the source image, and the method could accurately process the boundary of the focusing and nonfocusing regions. Compared with traditional methods, the use of CNN to fuse multifocus images is more advantageous. The design of fusion rules is the main task in multifocus image fusion, while CNN does not require manual design. The fusion rules can be obtained directly through network learning, and the generated fusion rules can be regarded as “optimal” to some extent; the main task of multifocus image fusion then becomes the design of the network structure. With the advent of CNN platforms such as Caffe [39], the design of the network is more convenient. The rapid development of GPUs makes it possible to apply large amounts of image data. Therefore, a method based on CNN is more likely to obtain high-quality fusion results.

## 3. Multifocus Deep Convolutional Neural Network

In recent years, the breakthrough of deep neural networks comes from deep convolutional neural networks. Convolutional neural networks represent a special case of artificial neural networks, which are inspired by animal visual cortex neural networks. Convolutional neural networks consist of continuous linear functions and nonlinear functions. The local characteristics of the convolution can effectively process the image, while the presence of a nonlinear function allows for more complex data representation. A CNN tries to learn the representation mechanism of features of image data at different levels of abstraction. Each convolution layer contains a certain number of feature maps, which correspond to the level of abstraction of features. The local receiving domain, shared weight, and subsampling are three basic structural concepts of CNNs.

**Figure 1:**
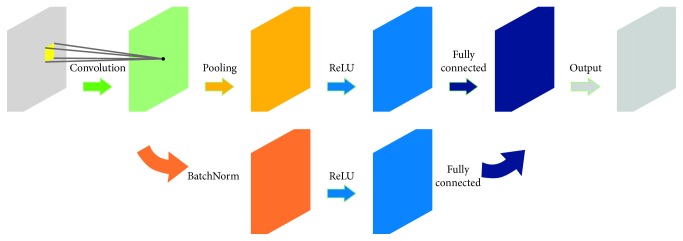
Basic building block of a deep convolutional network, including the input layer, convolution layer, pooling layer, active layer, fully connected layer, and output layer. To avoid overfitting, batch normalization is performed.

The correspondence between the input and output of the convolutional neural network is

(1) $$\begin{array}{c} y=F\left(\theta ,x\right)=f_{i}\left(\overset{n}{\underset{i=1}{\sum }}W_{i}x+b_{i}\right), \end{array}$$

where *x* is the input, *y* is the output, *W*_*i*_ is the convolution matrix of the *i*-th layer, and *b*_*i*_ is the deviation of the *i*-th layer. *f*_*i*_ is the excitation function and can be selected from several options, but the rectified linear unit (ReLU) is commonly used. *θ* is the set of all tunable parameters including *W*_*i*_ and *b*_*i*_. The goal of the CNN framework is to determine appropriate parameters to minimize the loss of experience:

(2) $$\begin{array}{c} \overset{K}{\underset{k=1}{\sum }}L\left(y_{k},F\left(\theta ,x_{k}\right)\right), \end{array}$$

where *x*_*k*_ and *y*_*k*_ represent the *k*-th input and output, respectively. *L*(·) denotes the Euclidean distance. The inverse error propagation algorithm is used to minimize equation (2). The basic structure of a CNN is composed of an input layer, a convolution layer, a pooling layer, the fully connected layer, and the output layer. To alleviate the overfitting problem, the number of internal variables can be reduced. Specifically, the training data are subdivided into specific small batch basic data units, a process known as batch normalization. The basic structure of a CNN is shown in Figure 1.

**Figure 2:**
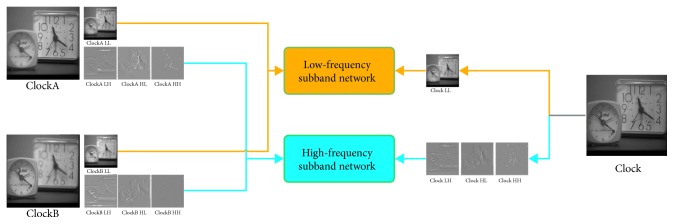
Deep convolutional neural network based on multifocus image fusion in the wavelet domain.

The algorithm principle of multifocus image fusion based on the CNN of the wavelet domain is shown in Figure 2. This paper primarily considers the case of only two source images. To process more than two multifocus images, we can concatenate them one-by-one. The approaches used in this paper mainly correspond to the wavelet domain transform, low-frequency subband network, and high-frequency subband network. One of the most important concepts in this paper is transforming the image into high-frequency and low-frequency subbands using wavelet transformation and using the CNN to train the high-frequency and low-frequency subbands of the image separately instead of directly using the source image for end-to-end training. Using wavelet transforms, the image is effectively decomposed, thereby making it easier to train deep networks.

### 3.1. Wavelet Transform

**Figure 3:**
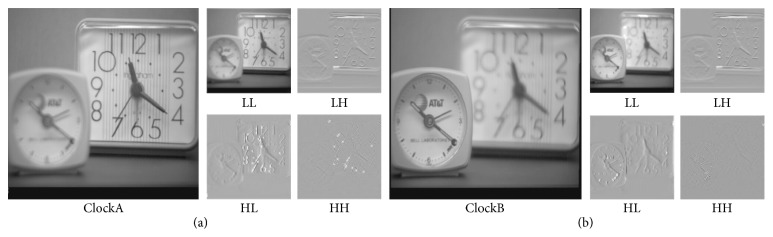
Source image wavelet transform. (a) ClockA wavelet transform. (b) ClockB wavelet transform.

The image fusion algorithm based on wavelet transform can be divided into two parts: wavelet transform and fusion rule. The fusion rules include the low-frequency subband fusion rules and high-frequency subband fusion rules, which determine the quality of the fused image. Therefore, most of the research is focused on the fusion rules. In this paper, we use a CNN to learn the fusion rules of low-frequency and high-frequency subbands. The wavelet transform is used to decompose the source image into a series of wavelet images of different frequencies: the low-frequency subband graph LL, which maintains the main information of the source image, and the high-frequency subband graphs LH, HL, and HH, which maintain the horizontal edge details of the source image, vertical edge detail, and diagonal edge detail, respectively. The wavelet includes the frequency of the transform and the finite duration and has the ability to localize the time-frequency. After the image is decomposed by the wavelet, it can be expressed as

(3) $$\begin{array}{c} \begin{array}{c} \left\{\begin{array}{c} C_{j+1}\left(m,n\right)=\underset{r\in Z}{\sum }\underset{c\in Z}{\sum }H_{r-2m}H_{c-2n}C_{j},\\ D_{j+1}^{H}\left(m,n\right)=\underset{r\in Z}{\sum }\underset{c\in Z}{\sum }G_{r-2m}H_{c-2n}C_{j},\\ D_{j+1}^{V}\left(m,n\right)=\underset{r\in Z}{\sum }\underset{c\in Z}{\sum }H_{r-2m}G_{c-2n}C_{j},\\ D_{j+1}^{D}\left(m,n\right)=\underset{r\in Z}{\sum }\underset{c\in Z}{\sum }G_{r-2m}G_{c-2n}C_{j}, \end{array}\right. \end{array} \end{array}$$

where *H*_*r*_ and *H*_*c*_ represent the high-pass filters and *G*_*r*_ and *G*_*c*_ represent the low-pass filters; *r* and *c* represent the rows and columns of the image, respectively; *C*_*j*+1_ represents the low-frequency part of the image; and *D*_*j*+1_^*H*^, *D*_*j*+1_^*V*^, and *D*_*j*+1_^*D*^, respectively, represent the edge details of the image in the horizontal, vertical, and diagonal directions. The wavelet decomposition of the image is shown in Figure 3.

### 3.2. High-Frequency Subband Network

#### 3.2.1. Network Structure

**Figure 4:**
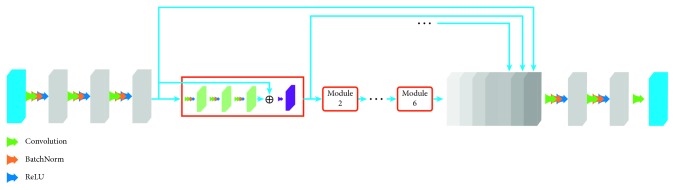
High-frequency subband network.

In this paper, a high-frequency subband network is trained according to the wavelet domain residual network, and its structure is shown in Figure 4. The high-frequency subband network designed in this paper is used to fuse the high-frequency information of the image. The wavelet is used to decompose the image, and each image can obtain three high-frequency components. The network structure of the high-frequency subband network consists of 24 convolution layers, followed by the batch normalization layer and ReLU layer. Batch normalization can effectively improve the learning efficiency of the neural network. In the first layer of convolution, 128 sets of 3 × 3 × 6 convolution kernels are used, and 128 sets of 3 × 3 × 128 convolution kernels are used in the subsequent convolutional layers. The high-frequency subband network consists of six modules, each consisting of one bypass connection and three convolutional layers. In addition, there is a channel connection layer in the network to stack the input of each module to allow the gradient to undergo back propagation on different paths and achieve faster end-to-end training. The batch normalization operation is also performed on the input of each module, which can effectively improve the learning efficiency of the next layer.

The greater the number of network layers is, the greater the likelihood of achieving high quality results is, subject to circumstances. The high-frequency subband network has a 24-layer convolution and can achieve sufficiently satisfactory results. However, as the number of layers increases, the cost of network training increases and the possibility of gradient dispersion and gradient explosions increases. To prevent this and accelerate convergence, based on the idea of residual learning, a bypass connection is used in each module and a batch normalization operation is performed for each module.

The choice of patch is a critical issue in network design. Choosing a 32 × 32 patch usually achieves satisfactory precision because the patch is sufficiently large to allow the use of more image content. However, for multifocus image fusion, it is usually inappropriate to select a 32 × 32 patch as it is more likely to contain focusing and defocusing regions, which will lead to undesirable results at the boundary of the fusion image. An 8 × 8 patch is too small to guarantee the accuracy of the fused image. After testing, 16 × 16 patches are selected to achieve the best results. Each patch comes from 16 × 16 square areas of six high-frequency images; thus, the total size of the patch is 16 × 16.

#### 3.2.2. Wavelet Loss Function

The high-frequency subband network aims at learning the mapping of the high-frequency components of the source image to high-frequency components of the fused image to realize the fusion of multifocus images. To obtain a satisfying high-frequency texture detail, we can use wavelet-based loss to help reconstruct the texture [40, 41]. This paper uses two wavelet-based losses: weighted wavelet domain MSE loss and texture loss. The MSE loss in the weighted wavelet domain is shown in the following equation:

(4) $$\begin{array}{c} l_{\text{wavelet}}\left(\hat{C},C\right)={\left|W^{1/2}⊙\left(\hat{C}-C\right)\right|}_{F}^{2}\\ =\overset{n}{\underset{I=1}{\sum }}\lambda_{I}{\left|{\hat{c}}_{i}-c_{i}\right|}_{F}^{2}\\ =\lambda_{1}{\left|{\hat{c}}_{1}-c_{1}\right|}_{F}^{2}+\overset{n}{\underset{I=2}{\sum }}\lambda_{I}{\left|{\hat{c}}_{i}-c_{i}\right|}_{F}^{2}, \end{array}$$

where *W*=(*λ*_1_, *λ*_2_,…, *λ*_*N*_) is the weight matrix that balances the different wavelet coefficients so that the training focuses on the texture with higher weight coefficients. *C*=(*c*_1_, *c*_2_,…, *c*_*N*_) and $\hat{C}=\left({\hat{c}}_{1},{\hat{c}}_{2},\ldots ,{\hat{c}}_{N}\right)$ represent the wavelet coefficients of the fused image and the wavelet coefficients of the input source image, respectively. ${\left\|{\hat{c}}_{1}-c_{1}\right\|}_{F}^{2}$ is used to capture the global topology information, which helps maintain training stability when approximating function *c*_1_ as the input. The following equation represents a texture loss function designed to prevent high-frequency wavelet coefficients from converging to zero:

(5) $$\begin{array}{c} l_{\text{texture}}=\overset{n}{\underset{i=k}{\sum }}\gamma_{i}\max\left(\alpha {\left|c_{i}\right|}_{F}^{2}+\varepsilon -{\left|c_{i}\right|}_{F}^{2},0\right), \end{array}$$

where *k* represents the starting index of the wavelet coefficients, and it penalizes the smaller wavelet coefficients. *γ*_*i*_ is the balance parameter, and *α* and *ε* are the relaxation parameters to ensure that the high-frequency wavelet coefficient remains nonzero, thereby avoiding detail degradation.

The two loss functions based on the wavelet domain together constitute the loss function of the high-frequency components in the high-frequency subband network. The unified loss function is expressed as

(6) $$\begin{array}{c} l_{\text{total}}=l_{\text{wavelet}}+\mu l_{\text{texture}}\\ =\overset{n}{\underset{i=1}{\sum }}\lambda_{i}{\left|{\hat{c}}_{i}-c_{i}\right|}_{F}^{2}+\mu \overset{n}{\underset{i=1}{\sum }}\gamma_{i}\max\left(\alpha {\left|c_{i}\right|}_{F}^{2}+\varepsilon -{\left|{\hat{c}}_{i}\right|}_{F}^{2},0\right), \end{array}$$

where *μ* is the equilibrium parameter. In this paper, we use the stochastic gradient descent (SGD) method and the error back propagation method to minimize the loss function. The initial learning rate is set to 0.01, and it gradually reduced to 10^−5^.

### 3.3. Low-Frequency Subband Network

#### 3.3.1. Network Structure

**Figure 5:**
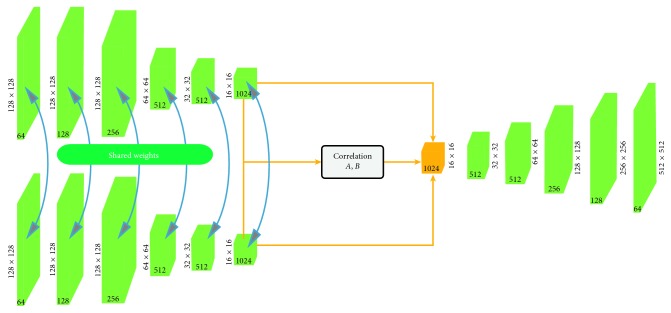
Low-frequency subband network.

The low-frequency subband network also adopts an end-to-end training method, and its structure is shown in Figure 5. The network structure pertains to the following three steps. (1) The wavelet is used to obtain two low-frequency images *I*_*A*_ and *I*_*B*_ from source image decomposition, and a Siamese encoder composed of convolutional layers is used to extract high-level semantic feature mapping *l*_*A*_ and *l*_*B*_. (2) Next, using a fusion layer, feature map fusion is performed at the pixel level to obtain a feature map *f* for multifocus image fusion. (3) Finally, given the connection of feature maps *l*_*A*_ and *l*_*B*_ and *f*, the low-frequency component of the fused image is obtained using a decoder consisting of deconvolution.

The first part of the Siamese encoder consists of two CNNs with shared weights. On the basis of the VGG network [42], the first 13 convolutional layers are retained, and the last fully connected layer is transformed into a 3 × 3 convolutional layer to obtain more feature mappings. Based on this, a low-frequency subband network is designed to be applied to image fusion. The Siamese encoder has 15 convolutional layers to extract the feature maps *l*_*A*_ and *l*_*B*_ of *I*_*A*_ and *I*_*B*_, respectively. Two low-frequency images of 128 × 128 are input, and two 1024-channel feature maps are output through the Siamese encoder, in which each layer is 16 × 16.

The second part is the fusion layer. To fuse the feature maps *l*_*A*_ and *l*_*B*_, the spatial correspondence and channel correspondence must be realized. When layers that need to be fused have the same resolution, it is easier to achieve spatial correspondence by performing stacking on one network. Channel correspondence is relatively difficult to achieve, and it is necessary to address the correspondence between channels in one network and channels in another network.

The connection function is defined as *y*_cat_=*f*_cat_(*l*_*A*_, *l*_*B*_). Stacking two feature maps at the same spatial positions *i* and *j* of channel *d*, we obtain

(7) $$\begin{array}{c} y_{\text{cat}}^{i,j,2d}=l_{A}^{i,j,d}y_{\text{cat}}^{i,j,2d-1}=l_{B}^{i,j,d}. \end{array}$$

A set of fusion filters *F* and deviation *b* are used to convolve fuse the data:

(8) $$\begin{array}{c} y_{\text{conv}}=y_{\text{cat}}\times F+b, \end{array}$$

where the size of the fusion filter *F* is 1 × 1 × 2.

The third part consists of the decoder. The input to the decoder is given by connecting the feature mappings *l*_*A*_ and *l*_*B*_ and their corresponding correlation mapping *f*. The convolution acts as an encoder that maps multiple inputs to a single output within the filter window, while deconvolution corresponds to a single input and multiple output decoders. The decoder corresponds to the encoder. There are five modules in the decoder; each module has one deconvolution layer and two convolution layers, and all the layers and deconvolution layers have one ReLU activation function. The decoder is a key part of the low-frequency subband network, and high-quality fused images can be obtained via deconvolution operations. The filter in the decoder section is not fixed but can be learned.

#### 3.3.2. Loss Function

Learning the mapping from a blurred image to a clear image requires updating the parameter *θ*(*ω*, *b*). {(*l*_*A*_^*i*^, *l*_*B*_^*i*^), *f* ^*i*^} is a set of *N* training sample pairs, where *l*_*A*_^*i*^ and *l*_*B*_^*i*^ represent a one-to-many focused image and *f* ^*i*^ represents a clear image. The loss function mean squared error (MSE) is minimized as

(9) $$\begin{array}{c} L\left(\theta \right)=\frac{1}{N}\overset{n}{\underset{i=1}{\sum }}{\left|F\left(x_{i}^{a},x_{i}^{b};\theta \right)-y_{i}\right|}_{2}^{2}. \end{array}$$

Similar to the high-frequency subband network, the low-frequency subband network also uses the SGD method and the error back propagation method to minimize the loss function.

## 4. Experiment and Analysis

### 4.1. Network Training

This paper used the Caffe framework [39] to design and implement a CNN network. The learning rate was initially set at 0.01, and it continuously reduced to 10^−5^. The momentum attenuation was 0.9, and the weight attenuation was 0.0005. The size of the patch was 16 × 16. The loss function of the high-frequency subband network was a unified loss function composed of two wavelet-based losses, the weighted wavelet domain MSE loss and texture loss. The loss function of the low-frequency subband network was the mean square error. Both subband networks used the SGD method and the error back propagation method to minimize the loss function.

All experiments reported in this paper were implemented in the following environment: Intel Core i7 CPU, GTX1080 GPU, 16G RAM, platform MATLAB2017b.

### 4.2. Training Set

**Figure 6:**
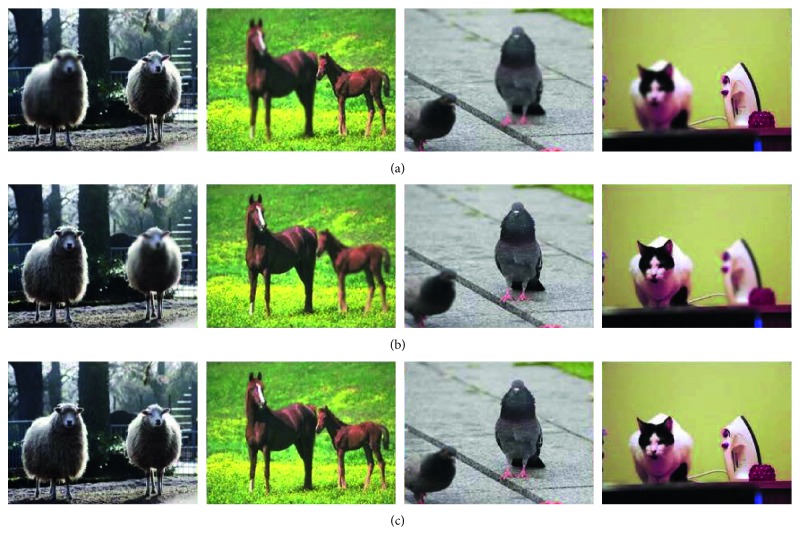
Training data set. (a) and (b) show a pair of blurred images, and (c) shows a clear image.

The training dataset was composed of 8000 high-quality natural images selected from the ILSVRC 2013 ImageNet dataset. For each image, a Gaussian filter was used to obtain a blurred version with different levels of blur. Specifically, the Gaussian filter sets the standard deviation from 2 to 7. The original image was processed using the Gaussian filter, and a set of images with different fuzzy regions was obtained. The first blurred image was obtained from the original clear image using a Gaussian filter, and the second blurred image was obtained from the first blurred image. Gaussian blurring was carried out for five different standard deviations to ensure that the trained network could address most fuzzy cases. A total of 8000 sets of images (8000 pairs of fuzzy images and 8000 clear images) were obtained from the ILSVRC 2013 dataset. Part of the training is shown in Figure 6.

### 4.3. Test Set

**Figure 7:**
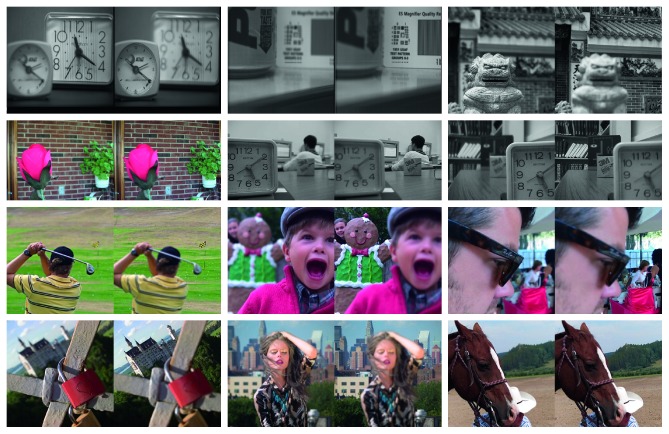
Partial test image.

To verify the effectiveness of the proposed wavelet-based deep convolutional network, we selected 40 pairs of images for testing. Among them, 20 pairs have been widely used in multifocus image fusion, and the other 20 pairs were selected from the Lytro dataset. Part of the test image is shown in Figure 7. The upper part shows the conventional test image and the lower part shows the image selected from the Lytro dataset.

### 4.4. Evaluation Indicators

Objective evaluation plays a vital role in image fusion. The quality of image fusion needs to be evaluated by quantitative scores of multiple indicators. In recent years, some objective performance indexes for multifocus image fusion have been proposed. This paper employs four indexes to evaluate the quality of image fusion.

(1) Mutual information (MI) [43] is used to evaluate the mutual information between the fused image and the source image: the mutual information is used to estimate the joint information between the source images *I*_*A*_ and *I*_*B*_ and the fused image *I*_*F*_: MI_*F*_^*A*,*B*^=*I*(*F*, *A*)+*I*(*F*, *B*). Here, *A*, *B*, and *F* are the normalized histograms of source images *I*_*A*_ and *I*_*B*_ and fused image *I*_*F*_, respectively. There are two unnormalized quantities in MI_*F*_^*A*,*B*^ that make it bias the source image with the highest entropy. To better evaluate the fused image, the mutual information is normalized:
  (10) $$\begin{array}{c} {\text{MI}}_{F}^{A,B}=2\left[\frac{I\left(F,A\right)}{H\left(F\right)+H\left(A\right)}+\frac{I\left(F,B\right)}{H\left(F\right)+H\left(AB\right)}\right], \end{array}$$
  where *H*(*A*), *H*(*B*), and *H*(*F*) are the entropies of *A*, *B*, and *F*, respectively.
(2) An average gradient- (AG-) based evaluation [44] is used to assess the extent to which a fused image obtains image detail from a source image: for a fused image of size *M* × *N*, AG is defined as
  (11) $$\begin{array}{c} \text{AG}=\frac{1}{\left(M-1\right)\left(N-1\right)}\overset{M-1}{\underset{m=1}{\sum }}\overset{N-1}{\underset{n=1}{\sum }}\frac{1}{4}\sqrt{{\left(\frac{\partial I\left(m,n\right)}{\partial m}\right)}^{2}{\left(\frac{\partial I\left(m,n\right)}{\partial n}\right)}^{2}}, \end{array}$$
  where (*m*, *n*) represents the coordinates of the image and (∂*I*/∂*m*) and (∂*I*/∂*n*) represent the horizontal and vertical gradients, respectively. A larger AG corresponds to a sharper edge of the image.
(3) Structural similarity index- (SSIM-) based evaluation [45]: the structural information saved in the fusion image is evaluated. The similarity in the local structures between images is used as the matching measure.

The structural similarity between the source images *I*_*A*_ and *I*_*B*_ and the fused image *I*_*F*_ is as follows:

(12) $$\begin{array}{c} Q\left(I_{A},I_{B},I_{F}\;|\;w\right)=\left\{\begin{array}{cc} \lambda \left(w\right)\text{SSIM}\left(I_{A},I_{F}\;|\;w\right)+\left(1-\lambda \left(w\right)\right)\text{SSIM}\left(I_{B},I_{F}\;|\;w\right), & \text{for SSIM}\left(I_{A},I_{B}\;|\;w\right)\geq 0.75,\\ \max\left\{\text{SSIM}\left(I_{A},I_{F}\;|\;w\right),\text{SSIM}\left(I_{B},I_{F}\;|\;w\right)\right\}, & \text{for SSIM}\left(I_{A},I_{B}\;|\;w\right)<0.75, \end{array}\right. \end{array}$$

where SSIM(*I*_*A*_, *I*_*B*_ | *w*), SSIM(*I*_*A*_, *I*_*F*_ | *w*), and SSIM(*I*_*B*_, *I*_*F*_ | *w*) represent the local structure similarities of source images *I*_*A*_ and *I*_*B*_, source image *I*_*A*_ and fused image *I*_*F*_, and source image *I*_*B*_ and fused image *I*_*F*_, respectively. *w* is the 7 × 7 sliding window. *λ*(*w*) denotes the local weight, and *Q*(*I*_*A*_, *I*_*B*_, *I*_*F*_) denotes the global mass:

(13) $$\begin{array}{c} \lambda \left(w\right)=\frac{s\left(I_{A}\;|\;w\right)}{s\left(I_{A}\;|\;w\right)+s\left(I_{B}\;|\;w\right)},\\ Q\left(I_{A},I_{B},I_{F}\right)=\frac{1}{\left|w\right|}\sum_{w\in W}Q\left(I_{A},I_{B},I_{F}\;|\;w\right), \end{array}$$

where *s*(*I*_*A*_ | *w*) and *s*(*I*_*B*_ | *w*) denote the respective variance values of *w*_*A*_ and *w*_*B*_. The closer the value of *Q*(*I*_*A*_, *I*_*B*_, *I*_*F*_) is to 1, the higher the quality of the fused image is.

(4) Metrics based on contrast enhancement and image fusion (CEIF) [46] are used for the evaluation of image fusion and contrast enhancement, and the definition of CEIF is as follows:
  (14) $$\begin{array}{c} \text{CEIF}=\frac{1}{M}\underset{\left(x,y\right)\in \Omega }{\sum }S\left(x,y\right)O\left(x,y\right), \end{array}$$
  where *S*(*x*, *y*) represents the amount of edge strength enhancement and *O*(*x*, *y*) represents the coincidence of (*x*, *y*) at the edge orientation:
  (15) $$\begin{array}{c} S\left(x,y\right)=\left\{\begin{array}{cc} \frac{1}{1+\exp\left(-\kappa \left(\left(s_{F}\left(x,y\right)-s_{A}\left(x,y\right)\right)/\left(s_{F}\left(x,y\right)+s_{A}\left(x,y\right)\right)\right)\right)}, & \text{if}\;\;s_{A}\left(x,y\right)>s_{B}\left(x,y\right),\\ \frac{1}{1+\exp\left(-\kappa \left(\left(s_{F}\left(x,y\right)-s_{B}\left(x,y\right)\right)/\left(s_{F}\left(x,y\right)+s_{B}\left(x,y\right)\right)\right)\right)}, & \text{otherwise,} \end{array}\right. \end{array}$$
  where *κ*=5.
  (16) $$\begin{array}{c} s_{i}\left(x,y\right)=\frac{\sqrt{e_{i}^{x}{\left(x,y\right)}^{2}+e_{i}^{y}{\left(x,y\right)}^{2}}}{s_{\max}},\;i\in \left\{I_{A},I_{B},I_{F}\right\}. \end{array}$$

Let

(17) $$\begin{array}{c} O\left(x,y\right)=\left\{\begin{array}{cc} \cos^{2}\left(\left|o_{F}\left(x,y\right)-o_{A}\left(x,y\right)\right|\right), & \text{if}\;\;s_{A}\left(x,y\right)>s_{B}\left(x,y\right),\\ \cos^{2}\left(\left|o_{F}\left(x,y\right)-o_{B}\left(x,y\right)\right|\right), & \text{otherwise}, \end{array}\right. \end{array}$$

where

(18) $$\begin{array}{c} o_{i}\left(x,y\right)=\;\;\tan^{-1}\left(\frac{e_{i}^{y}\left(x,y\right)}{e_{i}^{x}\left(x,y\right)}\right),\;i\in \left\{I_{A},I_{B},I_{F}\right\}. \end{array}$$

In equations (16) and (18), *e*_*i*_^*x*^(*x*, *y*) and *e*_*i*_^*y*^(*x*, *y*) are two filtering images of the directional Sobel operator, including edge components of direction *x* and direction *y*, respectively. The larger the CEIF is, the better the contrast enhancement and image feature preservation in the source image are.

### 4.5. Comparison with Multiple Fusion Methods

To verify the effectiveness of wavelet-domain-based deep convolutional networks in multifocus image fusion, the proposed method is compared with eight representative multifocus image fusion algorithms, namely, the method based on nonsubsampled contour wave (NSCT) transform [18], method based on sparse representation (SR) [47], method based on nondownsampled contour wave and sparse representation (NSCT-SR) [20], method based on guidance filtering (GF) [48], method based on multiscale weighted gradient (MWG) [9], density-based SIFT (DSIFT) method [12], pixel-by-pixel convolution neural network (PCNN) method [35], and deep convolutional neural network- (DCNN-) based method [31]. The NSCT-based, SR-based, and NSCT-SR-based methods belong to the transformation domain method, and the NSCT-SR method can overcome the defects of the NSCT-based and the SR-based methods. The GF-based, MWG-based, and DSIFT-based methods are spatial domain methods. In the Lytro dataset, we compare the proposed approach with a method based on joint subband learning (JSL) [11].

### 4.6. Qualitative Analysis

**Figure 8:**
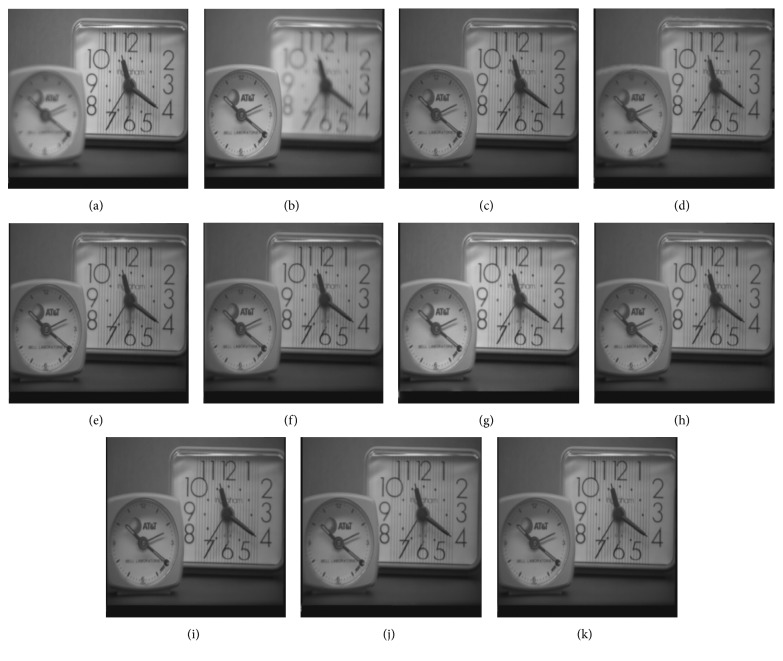
Clock source image and fusion image obtained different fusion methods. (a) ClockA. (b) ClockB. (c) NSCT. (d) SR. (e) NSCT-SR. (f) GF. (g) MWG. (h) DSIFT. (i) DCNN. (j) PCNN. (k) Proposed method.

**Figure 9 fig9:**
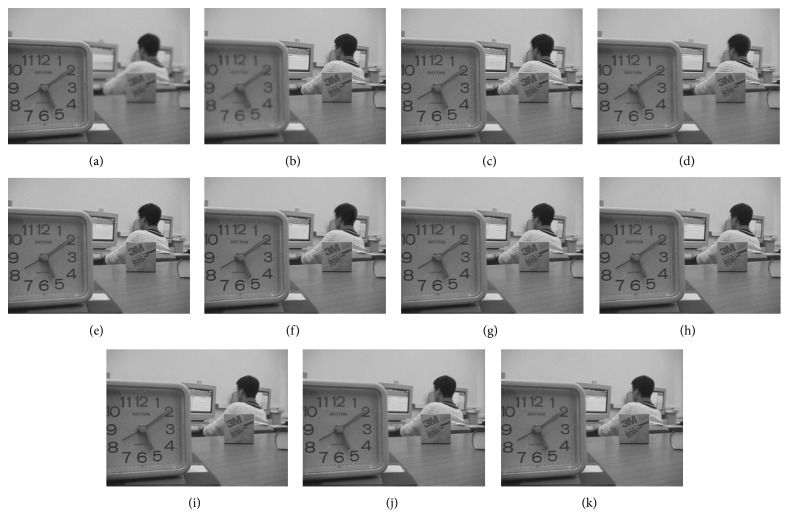
LAB source images and fusion images obtained using different fusion methods. (a) ClockA. (b) ClockB. (c) NSCT. (d) SR. (e) NSCT-SR. (f) GF. (g) MWG. (h) DSIFT. (i) DCNN. (j) PCNN. (k) Proposed method.

**Figure 10:**
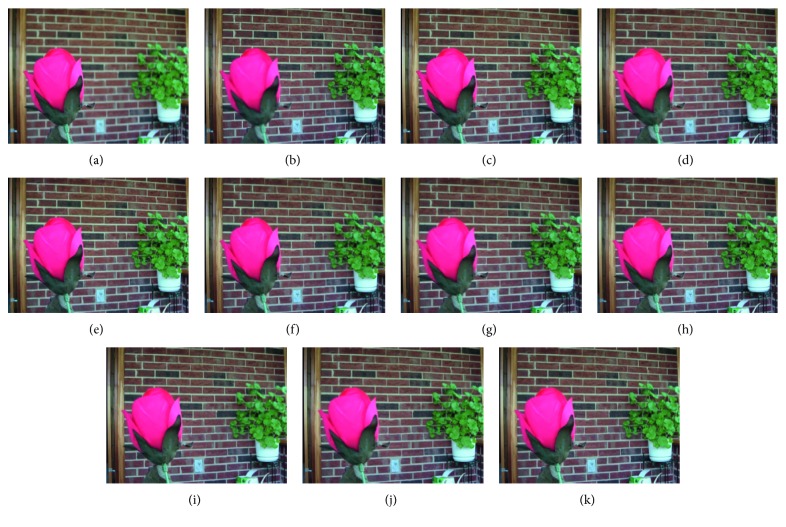
The flower source image and fused image obtained using different fusion methods. (a) FlowerA. (b) FlowerB. (c) NSCT. (d) SR. (e) NSCT-SR. (f) GF. (g) MWG. (h) DSIFT. (i) DCNN. (j) PCNN. (k) Proposed method.

Figures 8 [Fig fig9]–10 show the fused images obtained using different fusion methods. In other words, all of the fusion methods fuse the focus area of the source image. The SR, NSCT-SR, GF, DSIFT, and other methods enhance the fuzzy details while fusing the focus area of the source image. The proposed method also enhances the image details while fusing the source image, and the edge details enhanced by the proposed method are the clearest.

**Figure 11:**
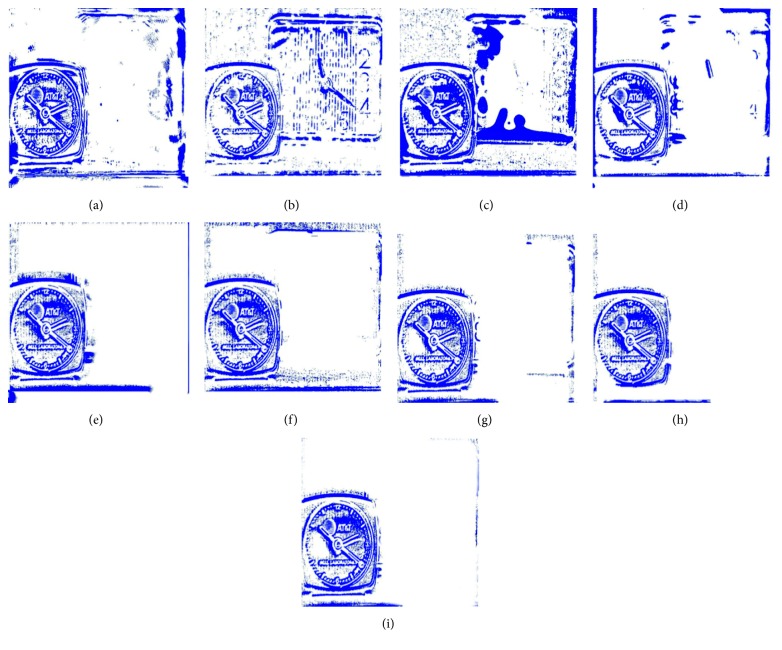
The difference between the fusion image and the source image ClockA. (a) NSCT. (b) SR. (c) NSCT-SR. (d) GF. (e) MWG. (f) DSIFT. (g) DCNN. (h) PCNN. (i) Proposed method.

**Figure 12:**
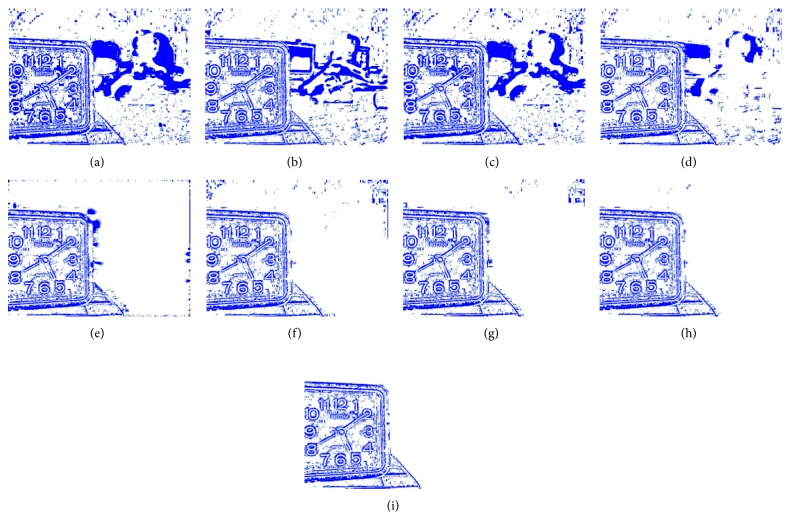
The difference between the fusion image and LabB of the source image. (a) NSCT. (b) SR. (c) NSCT-SR. (d) GF. (e) MWG. (f) DSIFT. (g) DCNN. (h) PCNN. (i) Proposed method.

Figure 8 shows the fusion of “clock” images using different fusion methods. Figure 9 shows the fusion of “lab” images using different fusion methods. For better comparison, Figure 11 shows the difference images obtained by subtracting ClockA source image from each fusion image, and Figure 12 shows the difference images obtained by subtracting LabB source image from each fusion image. In this paper, pseudo-colour enhancement is used to enhance the difference images for better contrast effect. As shown in Figures 11 and 12(a)–12(c), the fusion images obtained by the NSCT, SR, and NSCT-SR methods exhibit the worst effect and contain many unwanted artefacts, thereby indicating that the details of the image were not retained effectively. As shown in Figures 11 and 12(d)–12(f), considering the GF-based, MWG-based, and DSIFT-based methods, the fusion images obtained by the MWG method are generally of the highest quality, only slightly blurred at the focus and nonfocus boundary parts. The results are close to those obtained using the proposed method. The DSIFT-based method has some artefacts in the edge portion of the object. In the GF-based method, the processing of details in the partial regions is not sufficiently precise, such as at the boundary portion of the large clock in the clock image, and the boundary portion of the portrait in the lab image. As shown in Figures 11 and 12(g)–12(i), the quality of the fused images obtained by the DCNN and PCNN is the most similar to those obtained using the proposed method. The proposed method is notably superior to DCNN and PCNN in boundary processing.

In the difference image obtained by the proposed method for the clock image, only a small number of artefacts are present in the boundary part. Considering the difference image for the lab image, the proposed method achieved excellent results, with nearly no artefacts present. The PCNN-based method requires manual setting of the boundary between the focus region and the nonfocus region, which can achieve satisfactory fusion for some images, such as the clock image. However, the processing of small clock boundaries in the clock images is not sufficiently accurate. Partial artefacts exist in the difference images in the DCNN and PCNN methods. The fusion quality obtained by the proposed method in this paper is the best overall, and the performance is satisfactory in both the boundary region and the difference image.

**Figure 13:**
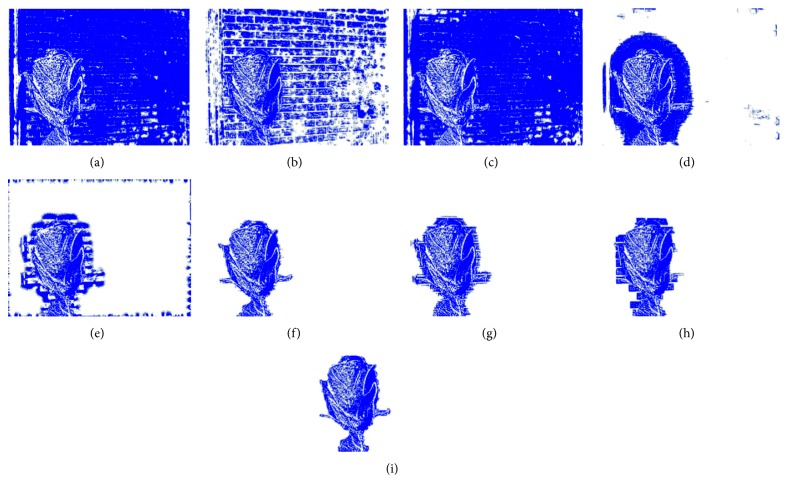
The difference between the fusion image and the source image FlowerB. (a) NSCT. (b) SR. (c) NSCT-SR. (d) GF. (e) MWG. (f) DSIFT. (g) DCNN. (h) PCNN. (i) Proposed method.

Figure 10 shows the fusion of “flower” images using different fusion methods.  Figure 13 shows the difference images obtained by subtracting flowerB source image from each fusion image. As shown in Figures 13(a)–13(c), the NSCT-based, SR-based, and NSCT-SR-based methods perform poorly in focusing, and there are a large number of unnecessary pixels in the difference images. The boundaries of the flowers are almost indistinguishable. As shown in Figures 13(d)–13(f), considering the GF-based, MWG-based, and DSIFT-based methods, the DSIFT-based method obtains the best quality of the fused image, which is closest to that obtained using the proposed method; however, in the case of the flower, the edge portion is not well blended. The GF-based method exhibits large boundary ranges in the focused and nonfocused boundary regions, and the MWG-based approach exhibits slight blurring in the focal and nonaggregated boundary regions. As shown in Figures 13(g)–13(i), the results obtained using the DCNN and PCNN are similar. The difference image of the DCNN contains a portion of the pixels that should have been subtracted in the boundary portion of the flower, causing the flower to be slightly deformed. Because PCNN needs to manually set the focus and nonfocus boundary areas, it is not possible to express flowers accurately. The difference image obtained using the proposed method represents the best result. After comprehensive consideration, the fused image obtained using the proposed method has better quality.

**Figure 14:**
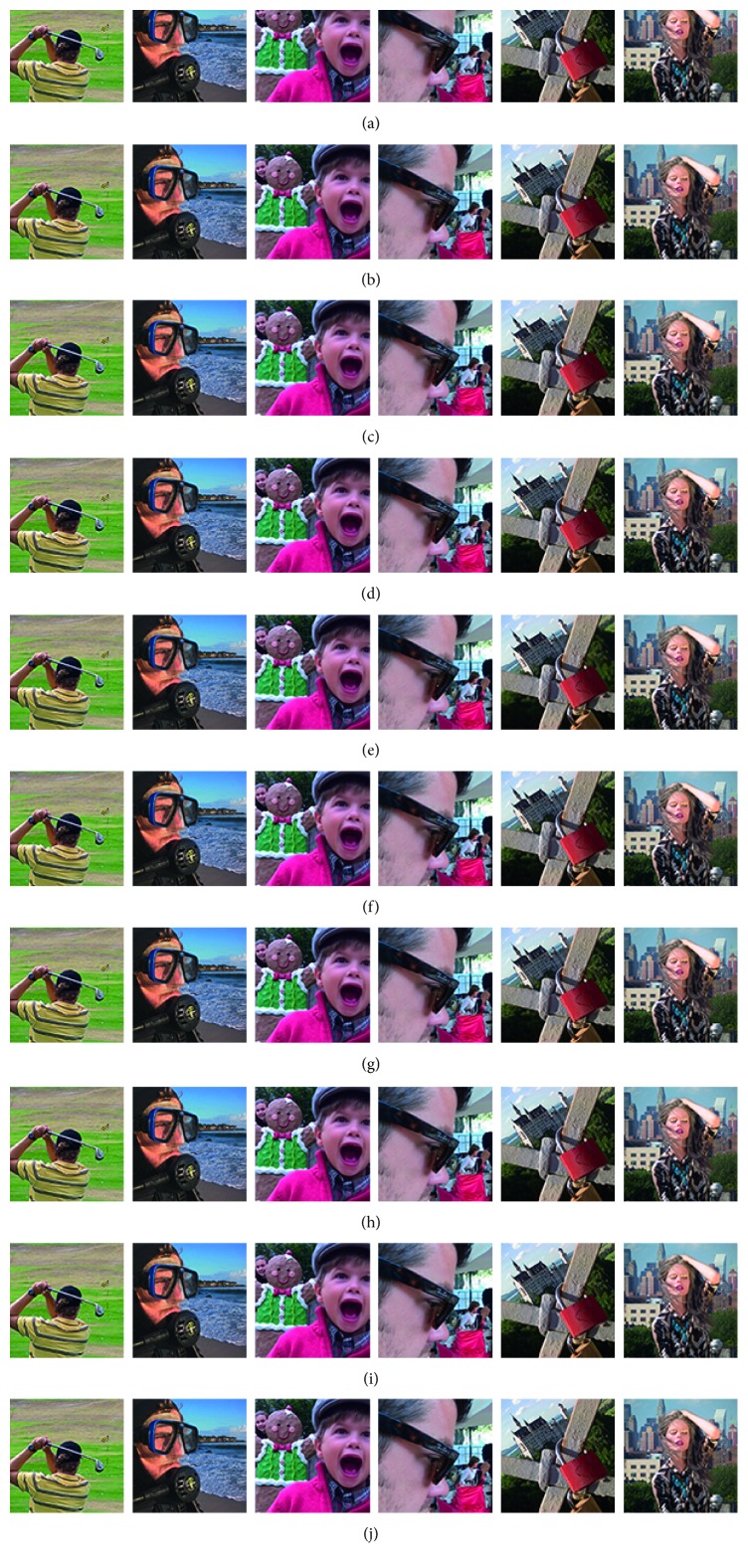
Fusion results for the Lytro dataset. (a) NSCT. (b) SR. (c) NSCT-SR. (d) GF. (e) MWG. (f) DSIFT. (g) DCNN. (h) PCNN. (i) JSL. (j) Proposed method.

**Figure 15:**
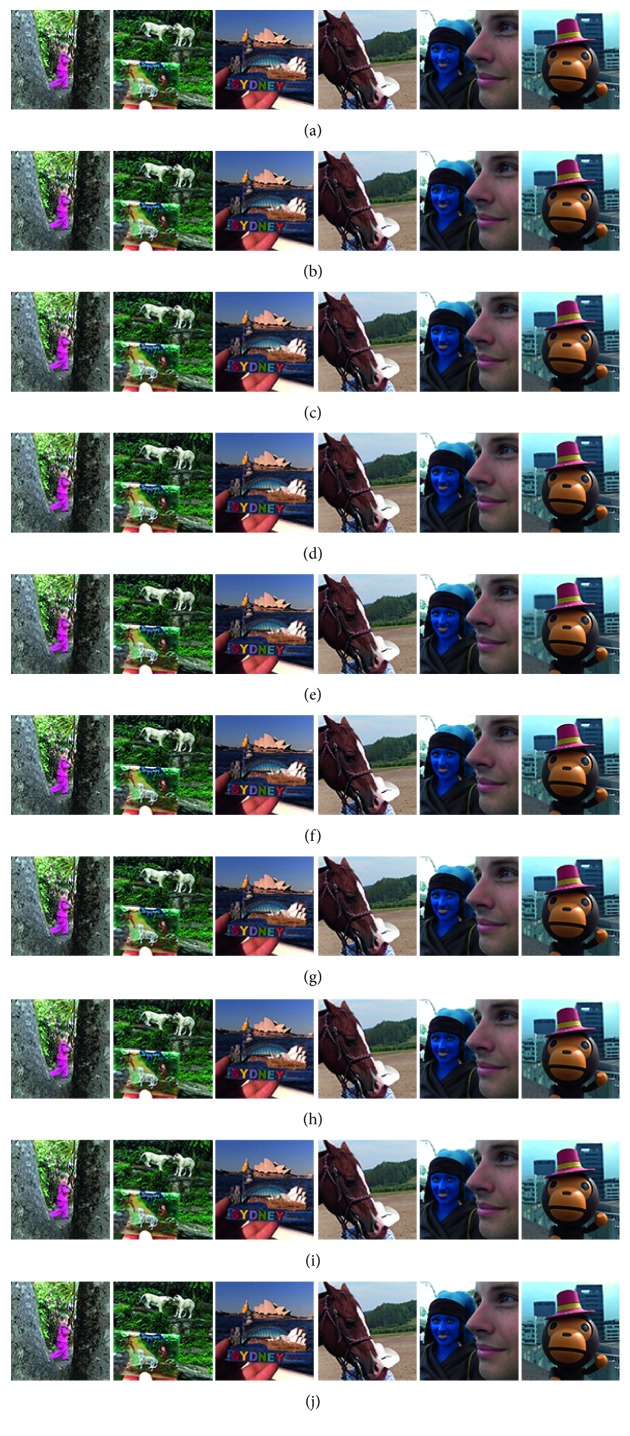
Fusion results for the Lytro dataset. (a) NSCT. (b) SR. (c) NSCT-SR. (d) GF. (e) MWG. (f) DSIFT. (g) DCNN. (h) PCNN. (i) JSL. (j) Proposed method.

**Figure 16:**
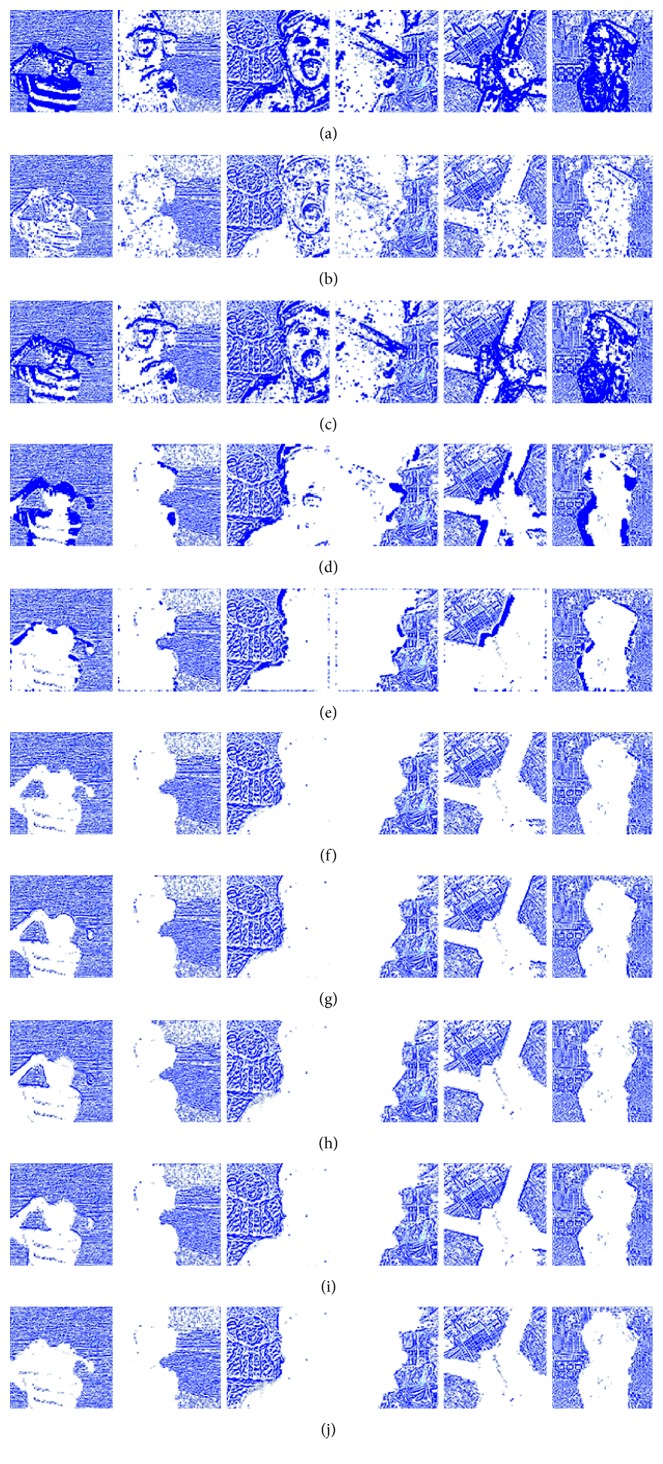
Difference images between fused and source images from the Lytro dataset. (a) NSCT. (b) SR. (c) NSCT-SR. (d) GF. (e) MWG. (f) DSIFT. (g) DCNN. (h) PCNN. (i) JSL. (j) Proposed method.

**Figure 17:**
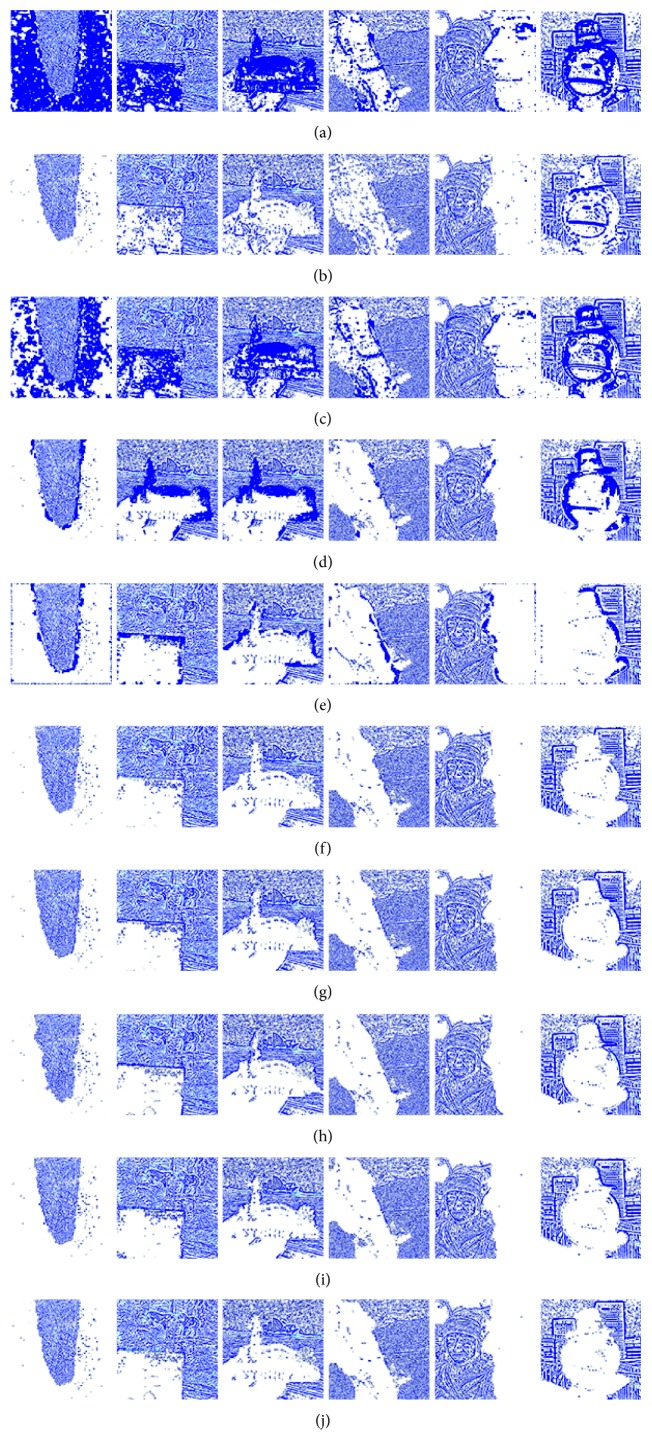
Difference images between fused and source images from the Lytro dataset. (a) NSCT. (b) SR. (c) NSCT-SR. (d) GF. (e) MWG. (f) DSIFT. (g) DCNN. (h) PCNN. (i) JSL. (j) Proposed method.

Figures 14 and 15 show the fusion results obtained for the Lytro dataset. Figures 16 and 17, respectively, show the difference images between the corresponding image and the source image shown in Figures 14 and 15. Considering only the fused image, it is difficult to distinguish the quality of fusion. The fusion quality can be better differentiated by using a large number of different images. In general, the quality of fused images obtained by the fusion method based on the spatial domain is better than that obtained by the fusion method based on the transformation domain. Considering only individual images, the fused image obtained by the transformation domain method is similar to that obtained by the spatial domain method. In column 1 of Figure 17(b), the fusion result obtained by the SR method is close to that obtained by the method based on the spatial domain. Moreover, the proposed method can obtain the optimal fusion result.

### 4.7. Quantitative Assessment

Using mutual information (MI), average gradient (AG), structural similarityindex- (SSIM-) based evaluation, and enhancement and image fusion (CEIF), the fusion results shown in Figures 6, 8, and 9 were quantitatively evaluated, and the evaluation results are presented in Tables 1 [Table tab2]–3, respectively. For better evaluation, we evaluated the images in the datasets of Figures 14 and 15 and took the average of their evaluations.  Table 4 presents the results of the images' evaluation.

**Figure 18:**
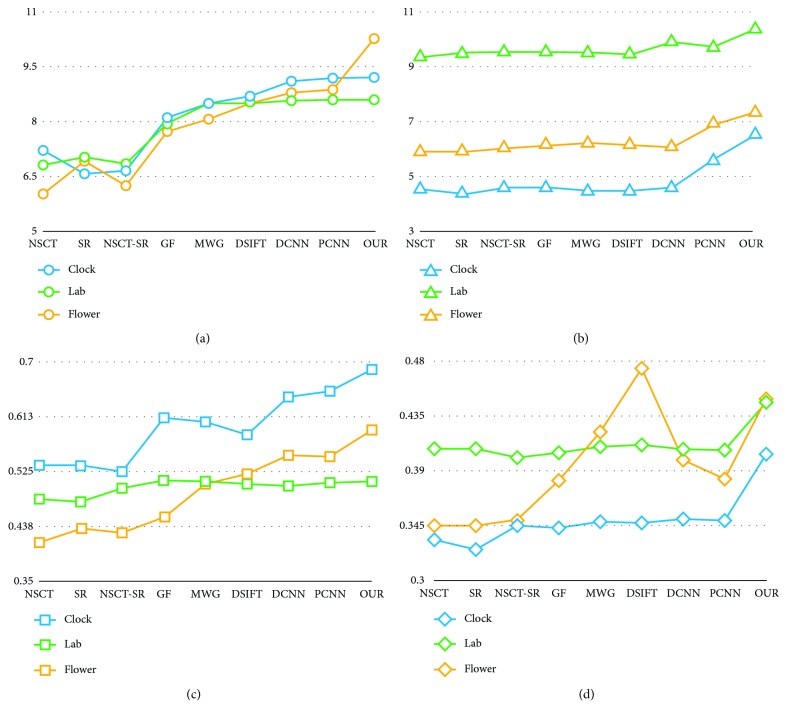
Evaluation result. (a) MI, (b) AG, (c) SSIM, and (d) CEIF.

**Figure 19:**
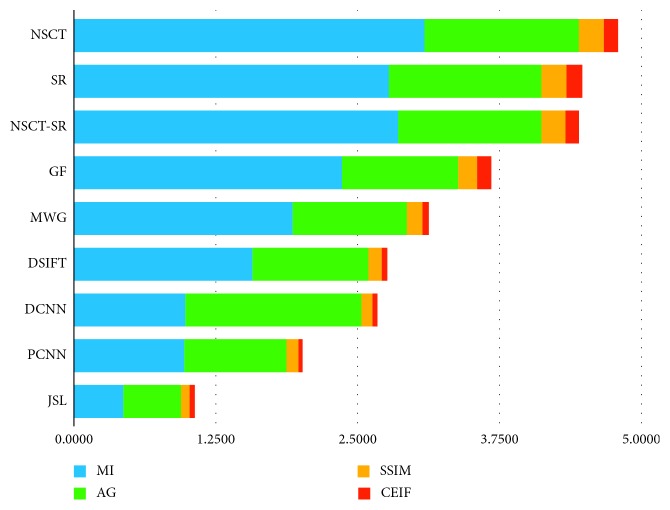
Proposed method is superior to other methods in terms of MI, AG, SSIM, and CEIF evaluation indicators.

According to the results of Tables 1 [Table tab2]–3, the proposed method exhibits better performance in the evaluation index used compared with that of other methods. Considering the CEIF evaluation presented in Table 3, the proposed method is slightly inferior than the DSIFT method. Considering the SSIM evaluation presented in Table 2, the proposed method is slightly inferior than the GF method. For other evaluations, the proposed method obtains the optimal results. The fused image obtained by the proposed method contains more details and has higher definition. The overall evaluation and analysis show that the proposed method exhibits better performance. Experimental results show that the source image is decomposed by the wavelet, and the low-frequency subband network and high-frequency subband network are, respectively, used for training and learning. The obtained fused image exhibits more details and better definition, and the processing between the focused region and the nonfocused region is more natural. In addition to the proposed method, the DCNN method and PCNN method can obtain satisfactory results. The method of deep learning is more advantageous than the traditional method. As seen from Table 4, the results obtained by the proposed method are the best, overall.  Figure 18 shows the results of evaluation of different fusion algorithms as per the MI, AG, SSIM, and CEIF evaluation indicators.  Figure 19 shows the extent to which the proposed method outperforms other methods in terms of the MI, AG, SSIM, and CEIF evaluation indicators.

## 5. Conclusions

By using a wavelet transform, a source image and sharp image are decomposed to obtain high-frequency and low-frequency information. This paper proposes a deep convolutional neural network based on wavelet transform. The high-frequency subband network is used to learn the mapping of high-frequency information of the source image to the high-frequency information of the clear image. The low-frequency subband network is used to learn the mapping of low-frequency information of the source image to the low-frequency information of the clear image. The high-frequency subband network uses batch normalization to speed up the convergence and uses residual learning to avoid gradient explosion and gradient dispersion. The key to the low-frequency subband network lies in the fusion layer, which determines the fusion quality of the low-frequency information. In this paper, the effectiveness of the wavelet-based deep convolutional network was verified. Experiments show that, for the case of the multifocus image fusion, the fusion results obtained by the proposed method exhibit better visual effects, and excellent results are obtained in terms of the evaluation indexes.

The wavelet transform used in this paper is the simplest wavelet transform. In future work, we will attempt to use different wavelet transforms, such as contour waves. In this paper, the characteristics of the wavelet are used to decompose the image through multiple channels to obtain more decomposed information; however, there is still room for improvement of the network structure and fusion scheme. For example, the patch size is set as 16 × 16 because it has the best fusion quality. However, larger size contains more information; thus, better fusion rules can be explored to adopt a larger patch size.

## Figures and Tables

**Table 1:** Quality evaluation of multifocus image “clock.”

	MI	AG	SSIM	CEIF
NSCT	7.2109	4.5281	0.5365	0.3342
SR	6.5797	4.3393	0.5337	0.3250
NSCT-SR	6.6648	4.5471	0.5268	0.3451
GF	8.0823	4.5768	0.6115	0.3431
MWG	8.4864	4.4446	0.6049	0.3482
DSIFT	8.6848	4.4438	0.5831	0.3474
DCNN	9.0994	4.5587	0.6452	*0.3507*
PCNN	*9.1897*	*5.5749*	*0.6543*	0.3495
OUR	**9.2079**	**6.5197**	**0.6889**	**0.4038**

The two best results are indicated in italics and bold.

**Table 2 tab2:** Quality assessment of multifocus image “lab.”

	MI	AG	SSIM	CEIF
NSCT	6.8302	9.3549	0.4831	0.4080
SR	7.0283	9.4561	0.4778	0.4088
NSCT-SR	6.8736	9.5427	0.4986	0.4004
GF	7.9061	9.5579	**0.5122**	0.4043
MWG	8.5083	9.5019	0.5096	0.4103
DSIFT	8.5154	9.4468	0.5049	*0.4112*
DCNN	8.5826	*9.8892*	0.5018	0.4079
PCNN	*8.5987*	9.7017	0.5075	0.4074
OUR	**8.6348**	**10.3541**	*0.5098*	**0.4462**

The two best results are indicated in italics and bold.

**Table 3:** Quality assessment of multifocus image “flower.”

	MI	AG	SSIM	CEIF
NSCT	6.0146	5.8791	0.4127	0.3451
SR	6.9018	5.9013	0.4345	0.3449
NSCT-SR	6.2435	6.0347	0.4277	0.3495
GF	7.7038	6.1339	0.4532	0.3821
MWG	8.0563	6.2189	0.5045	0.4219
DSIFT	8.5114	6.1253	0.5233	**0.4744**
DCNN	8.8071	6.0529	*0.5521*	0.3989
PCNN	*8.9057*	*6.8835*	0.5483	0.3835
OUR	**10.2569**	**7.3194**	**0.5924**	*0.4489*

The two best results are indicated in italics and bold.

**Table 4:** Lytro dataset quality assessment. The evaluation average of the images in Figures 14 and 15 are presented.

	MI	AG	SSIM	CEIF
NSCT	7.1563	7.3856	0.6729	0.3128
SR	7.4729	7.4037	0.6605	0.3003
NSCT-SR	7.3894	7.4869	0.6772	0.3185
GF	7.8835	7.7255	0.7154	0.3251
MWG	8.3194	7.7438	0.7461	0.3937
DSIFT	8.6741	7.7258	0.7658	0.3987
DCNN	9.2638	7.1983	0.7877	0.4032
PCNN	9.2741	7.8473	0.7815	*0.4047*
JSL	*9.8144*	*8.2385*	*0.8119*	0.4006
OUR	**10.2471**	**8.7502**	**0.8838**	**0.4473**

The two best results are marked with italics and bold.

## Data Availability

All data in this article are derived from publicly available datasets on the Internet.
